# Understanding the loss-of-function in a triple missense mutant of DNA polymerase β found in prostate cancer

**DOI:** 10.3892/ijo.2013.2022

**Published:** 2013-07-19

**Authors:** CHANGLONG AN, WILLIAM A. BEARD, DESHENG CHEN, SAMUEL H. WILSON, NICK M. MAKRIDAKIS

**Affiliations:** 1 Department of Epidemiology and Tulane Cancer Center, Tulane University, New Orleans, LA 70112, USA;; 2 Laboratory of Structural Biology, NIEHS, National Institutes of Health, Research Triangle Park, NC 27709, USA

**Keywords:** DNA repair, polymerase, enzyme activity, expression analysis

## Abstract

Human DNA polymerase (pol) β is essential for base excision repair. We previously reported a triple somatic mutant of pol β (p.P261L/T292A/I298T) found in an early onset prostate tumor. This mutation abolishes polymerase activity, and the wild-type allele was not present in the tumor, indicating a complete deficiency in pol β function. The effect on polymerase activity is unexpected because the point mutations that comprise the triple mutant are not part of the active site. Herein, we demonstrate the mechanism of this loss-of-function. In order to understand the effect of the individual point mutations we biochemically analyzed all single and double mutants that comprise the triple mutant. We found that the p.I298T mutation is responsible for a marked instability of the triple mutant protein at 37°C. At room temperature the triple mutant’s low efficiency is also due to a decrease in the apparent binding affinity for the dNTP substrate, which is due to the p.T292A mutation. Furthermore, the triple mutant displays lower fidelity for transversions *in vitro*, due to the p.T292A mutation. We conclude that distinct mutations of the triple pol β mutant are responsible for the loss of activity, lower fidelity, and instability observed *in vitro*.

## Introduction

Human DNA polymerase (pol) β is the primary polymerase involved in base excision repair (BER) an essential repair pathway that removes oxidized and alkylated bases from DNA (1). Its small size and monomeric nature make it an attractive candidate for biochemical and kinetic analysis (2). DNA polymerase β has also been suggested to be involved in DNA gap-filling reactions during meiotic synapsis (3), the repair of double-strand DNA breaks during non-homologous end joining (4), nucleotide excision repair of bulky DNA lesions (5,6) and replication (7). Targeted disruption of pol β in mice results in neonatal lethality, growth retardation, and apoptotic cell death in the developing nervous system suggesting a role for pol β in neurogenesis (8).

In contrast to the other human DNA polymerases, the availability of a high-resolution crystal structure of pol β in various liganded states provides a foundation to identify functionally important residues for mechanistic studies, as well as to interpret kinetic results with site-directed mutants. Furthermore, pol β shares many structural and mechanistic features with other DNA polymerases of known structure. For example, the mechanism of DNA polymerization follows an ordered binding of substrates to the enzyme, with the DNA template binding first (9). These attributes make pol β an excellent model for biochemical study of DNA synthesis and fidelity.

DNA polymerase β lacks a proofreading exonuclease domain, but encompasses two main domains: i) an amino-terminal 8-kDa lyase domain (responsible for the removal of the 5′-deoxyribose phosphate intermediate formed during BER) and ii) a carboxyl-terminal 31-kDa polymerase domain (10). The polymerase domain (residues 91–335) can be further separated into three functionally distinct subdomains, which correspond to the palm (C, catalytic), thumb (D, duplex DNA binding) and fingers (N, dNTP selection or nascent base pair binding) subdomains, according to the nomenclature that uses the architectural analogy to a right hand (11).

**Figure 1.**
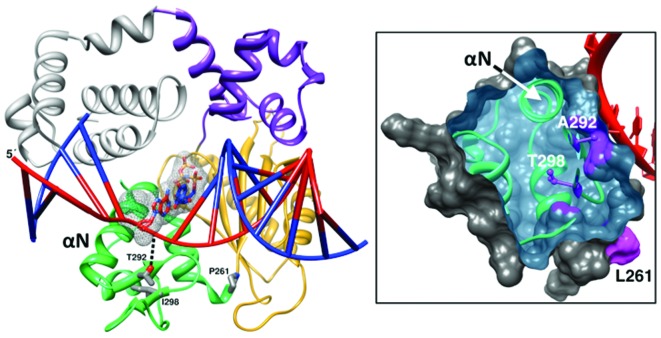
Position of the variant residues of the triple mutant in the structure of the DNA polymerase β ternary substrate complex. Left panel, a ribbon representation of pol β illustrating the polymerase (colored) and amino-terminal lyase (grey) domains (PDB code 2FMS). The polymerase domain is composed of three subdomains: D, purple; C, gold; and N, green. These correspond to the thumb, palm, and fingers subdomains of DNA polymerases that utilize an architectural analogy to a right-hand, respectively. The single-nucleotide gapped DNA is illustrated in a ladder representation with a red template strand and blue primer and downstream DNA strands. The 5′-end of the template strand is indicated. The nascent base pair (templating nucleotide and incoming nucleoside triphosphate) is shown in a stick representation with a mesh surface. The three residues (Pro261, P261; Thr292, T292; and Ile298, I298) of the N-subdomain altered in the triple mutant and α-helix N are shown. Right panel, the molecular surface of the N-subdomain is clipped to expose the internal position of the altered Thr298 (T298) and the surface exposed Ala292 (A292). The altered residues are shown in magenta as ball-and-sticks and their contribution to the surface is also shaded in magenta. Although the residue cannot be seen in this view, the surface contribution of Leu261 (L261) is indicated. The altered side chains were modeled based on probable rotamers from the Dunbrack library (36) and exhibited no clashes with nearby residues. The red template strand in the vicinity of the N-subdomain is also shown and α-helix N that interacts with the nascent base pair is labeled. The molecular images were produced in Chimera (35).

Comparisons of pol β structures in various liganded states have shown that upon dNTP binding to the binary (pol β/DNA) complex, the N-subdomain rotates from an open to closed state sandwiching the nascent base pair (dNTP-templating nucleotide) between α-helix N and the primer terminus base pair (12,13). These structures have also shown that productive binding of pol β to both gapped and nicked DNA requires a 90° bend in the DNA template strand at the 5′-phosphodiester linkage of the templating residue. The bend allows residues of the N-subdomain to interact with the nascent base-pair in the closed conformation (Fig. 1). Various mutagenesis studies have shown that the pol β/dNTP contacts produced by this bend are important for both polymerization efficiency and fidelity (reviewed in ref. 2).

Mutations in the pol β gene are commonly found in tumor tissues (14) and in several instances, these alterations are associated with diminished polymerase fidelity (15), catalytic activity (16), and increase in cellular transformation (17). By screening the complete coding region of the pol β gene in 26 prostate cancer tissues, we identified 20 somatic mutations, nine of them missense (18). Subsequent biochemical analysis of all missense pol β mutations demonstrated much smaller changes in enzyme activity for all mutants compared to the triple mutant, p.P261L/T292A/I298T, which had dramatically decreased activity (19). The pol β triple mutant was identified in an early-onset prostate cancer patient (18). No normal allele was present in the patient’s tumor, unlike the adjacent normal prostate that was wild-type, suggesting a clonal event during tumor evolution. In order to appreciate the significance of the triple mutant, it is essential to understand the molecular mechanism of the effects of the mutations. In addition, information about the structure-activity relationships of the mutations will enhance the understanding of the pol β polymerase activity.

Crystallographic analyses provide insight into the potential importance of the residues altered in the triple mutant. Threonine-292 (Thr292) is a surface exposed residue that is not part of the active site, but hydrogen bonds with the template backbone immediately upstream of the coding templating nucleotide (Fig. 1) (20). Alanine substitution would abolish this hydrogen bonding interaction, and thus may affect template binding, activity and/or fidelity. Proline-261 (Pro261) is situated at the boundary between the C- and N-subdomains that reposition themselves in response to nucleotide binding. Isoleucine-298 (Ile298) is distant from the active site and appears to form packing interactions in the mobile N-subdomain (Fig. 1). Threonine substitution would result in a buried side chain with hydrogen bonding capacity (Fig. 1, right panel). Thus the p.I298T substitution may alter the folding and/or stability of the N-subdomain. Given these relatively mild consequences expected by the single point mutations that comprise the triple mutant, the complete loss of pol β activity observed previously was unexpected.

We have constructed, purified and biochemically analyzed all single and double mutant variants *in vitro* that comprise the triple mutant in order to understand this apparent paradox regarding the activity of pol β. Herein we demonstrate that the loss of function exhibited by the triple mutant can be separated into distinct enzyme activity and enzyme stability changes, mainly afforded by the p.T292A and p.I298T point mutations, respectively.

## Materials and methods

### Bacterial strains and growth conditions

The strain BL21 DE3 was used for protein expression. *E. coli* DH5α, BL21 (DE3) and recombinant *E. coli* harboring *pol* β genes were cultured in LB medium containing kanamycin (50 *μ*g/ml) when appropriate.

### Construction of pol β variants

Wild-type (WT) *pol* β was obtained from J. Sweasy at Yale University. The mutants were obtained by the Stratagene Quick-change Site-Directed Mutagenesis kit according to the protocol of the manufacturer using the pET28a(+)-WT bacterial expression vector as a template (21). Successful mutagenesis was confirmed by DNA sequencing with BigDye chemistry on a 3100 ABI sequencer (Perkin-Elmer).

### Expression and purification of mutant enzymes

*E. coli* strain BL21 (DE3) carrying pET-28a(+)/pol β were grown at 37°C in LB medium containing 50 *μ*g/ml kanamycin with 1 mM IPTG. The cells were harvested by centrifugation, resuspended in 40 mM Tris, pH 8.0, 500 mM NaCl, 10 mM imidazole, and Protease Inhibitor Cocktail (as recommended by the manufacturer; Sigma). Resuspended cells were lysed by sonication. Extracts were cleared by centrifugation (15,000 rpm, 15 min at 4°C), and then loaded onto HisTrap FF crude Kit according to the manufacturer’s instructions (GE Healthcare). Proteins were eluted with 500 mM imidazole in 0.5 M NaCl. The elutants were loaded onto a HiTrap SP HP column (GE Healthcare). The column was washed with 100 mM NaCl and proteins were eluted with 2 M NaCl and stored at −80°C in 50 mM Tris, pH 8.0, 1 mM EDTA, 2 M NaCl, 10% glycerol and protease inhibitors as above (22,23). We then estimated enzyme homogeneity based on Coomassie Blue-stained SDS-PAGE gels. All proteins were quantified by Bradford protein assay (Sigma). This quantification together with the % homogeneity assessed by Coomassie Blue-stained gels (above) allowed us to quantify the enzyme amounts for each mutant (used in calculating Kcat below).

### Western blot analysis

Expressed His-tagged proteins were identified by western blot analysis (24). Proteins were electrophoresed in a 12% SDS-PAGE gel and transferred to a polyvinylidenedifluoride membrane (Thermo Scientific). Blots were blocked by 5% non-fat dry milk in Tris-buffered saline Tween-20 (0.1% Tween-20) and incubated with anti-His Tag antibody (Sigma) according to the manufacturer’s protocol. For detection, we used IRDye 800CW goat anti-rabbit IgG (LI-COR Biosciences) and the Odyssey imaging system (LI-COR Biosciences).

### DNA substrate

All oligonucleotides were synthesized and high-pressure liquid chromatography-purified by Integrated DNA Technologies. A 20-mer primer (5′-GCA GGA AAG CGA GGG TAT CC-3′) and 20-mer downstream oligonucleotide (5′-ACA AAG TCC AGC GTA CCA TA-3′) were annealed to a 46-mer template (5′-TAT GGT ACG CTG GAC TTT GTG GGA TAC CCT CGC TTT CCT GCT CCT G-3′) to generate a one-nucleotide gapped DNA substrate with a templating guanine (25). The 20-mer primer was 5′-labeled with [γ-^32^P]-ATP (3,000 Ci/mmol; Perkin-Elmer) using T4 polynucleotide kinase (US Biochemical Corp.) according to the manufacturer’s protocol. The 5′-^32^P-labeled primer was then purified from unincorporated label by a Microspin™ G-50 (GE Healthcare) column. The downstream oligonucleotide was 5′-phosphorylated by Integrated DNA Technologies. The oligonucleotides were annealed at a primer:template:downstream oligonucleotide molar ratio of 1:1.2:1.3 in 50 mM Tris, pH 8.0, 250 mM NaCl, in order to create a single nucleotide gap. The mixture was incubated at 95°C for 5 min, slow cooled to 50°C over 30 min, and incubated at 50°C for 20 min and then transferred to ice. Annealing of primer was confirmed on an 18% polyacrylamide (acrylamide/bis-acrylamide: 29:1) native gel followed by autoradiography as described (26,27).

### Protein stability assay

Protein stability was assessed by incubating pol β for varying lengths of time (3, 6, 9 and 12 min) at 37°C or room temperature (RT, 22°C). All reactions (20 *μ*l) were performed in 50 mM Tris-Cl, pH 8.0, 10 mM MgCl_2_, 2 mM DTT, 20 mM NaCl, 0.2 mg/ml BSA, 2.5% glycerol with 40 nM pol β and 50 nM DNA. All concentrations refer to the final concentration after mixing. The reaction mixtures were pre-incubated in the absence of dCTP and reactions were initiated by the addition of 12.8 *μ*M dCTP. After incubation for 2 min at 37°C and 6 min at RT, the reactions were quenched by adding 20 *μ*l of formamide loading buffer (900 *μ*l formamide, 22.2 *μ*l 0.5 M EDTA, pH 8.0 and 77.8 *μ*l water) and boiled for 10 min, and then transferred to ice. Products were resolved on a 15% polyacrylamide (acrylamide/bis-acrylamide: 29:1) gel containing 7 M urea. Gels were dried and the products were quantified with a PhosphorImager (Molecular Dynamics).

### Kinetic characterization

The conditions of all incorporation reactions were the same as those described above for the protein stability assay. Kinetic reactions were performed at 37°C for stable variants and at RT for all variants. To determine *K*_m,dNTP_, the reaction mixtures contained 2.5 nM purified pol β for correct incorporation and 50 nM pol β for incorrect incorporation with 50 nM annealed DNA substrate. All reactions were performed by first pre-incubating the DNA substrate with pol β for 3 min without dNTPs. Reactions were initiated by the addition of a single dNTP (0.1–2,000 *μ*M) and incubated for 2 min at 37°C (for stable variants) and for 6 min at RT (for all variants). For K_m,DNA_ determinations, RT reactions contained 20 nM WT or T292A pol β and 40 nM triple mutant; at 37°C, 8 nM enzyme was used. Reactions were initiated by addition of enzyme mixtures to annealed single-nucleotide gapped DNA substrate (0.01–3.2 *μ*M) and incubated for 4 min at RT or 37°C. The template nucleotide in the gap was deoxyguanosine and the dCTP concentration was 100 *μ*M. After incubation, the reactions were quenched as described above for the protein stability assay and quantified as above to obtain the percentage of product formed. Time courses were linear for the chosen enzyme concentration and time interval.

### Data analysis

The kinetic data were extracted from Lineweaver-Burk plots. We determined the values of *k*_cat_ and *K*_m,dNTP_ from trend line equations calculated from these plots with Microsoft Excel software (Microsoft). Apparent *k*_cat_ was calculated from *V*_max_, where *k*_cat_ = *V*_max_/(apparent enzyme). The apparent enzyme concentration was estimated from total protein. Fidelities for misinsertion reactions were calculated using the following equation: fidelity = [(*k*_cat_/*K*_m,correct_) + (*k*_cat_/*K*_m,incorrect_)]/(*k*_cat_/*K*_m,incorrect_).

## Results

### Triple mutant stability

**Figure 2.**
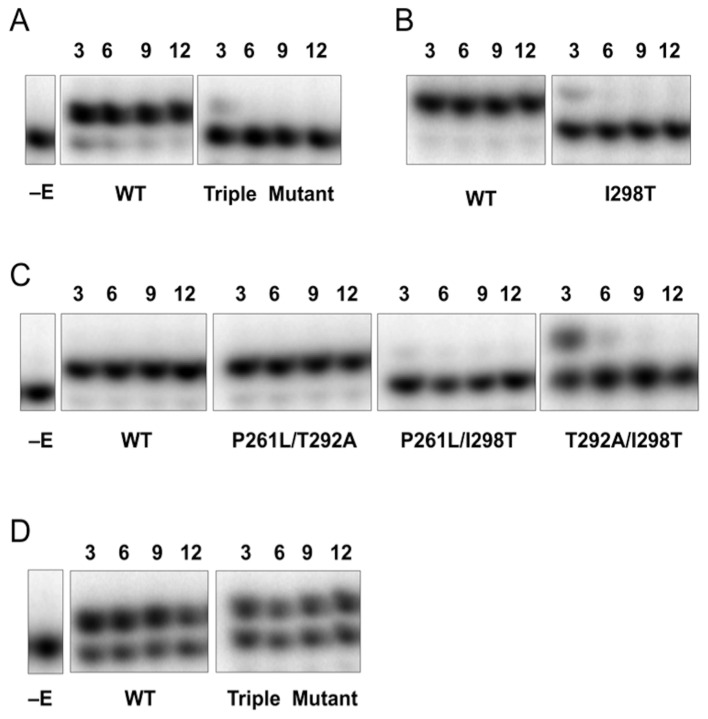
Stability of the DNA polymerase β variants. The stability of the pol β variants was assessed by activity after incubation at 37°C (panels A–C) or room temperature (22°C, panel D) for various lengths of time as described in Materials and methods. The panels show autoradiographes of gels depicting substrate (faster migrating band) and products (slower migrating band). The position of the ^32^P-labeled substrate band without extension (−E) is shown at the left. The stability of the triple mutant is compared to that of wild-type (WT) enzyme in panels A and D. The stability of p.I298T and the double mutants (p.P261L/T292A, p.P261L/I298T, and p.T292A/I298T) are compared to WT in panels B and C, respectively. The pre-incubation time intervals (min) are indicated above the respective lanes and refer to the period that enzyme and DNA were incubated before initiating the reaction with dCTP.

Variants of a triple mutant of human pol β identified previously in prostate cancer tissues (18) were obtained by site-directed mutagenesis. The WT, triple mutant (p.P261L/T292A/I298T), and all single and double mutant variants of pol β that comprise the triple mutant were expressed in *E. coli* and purified as described in Materials and methods. After purification, WT and the variants of pol β were analyzed by SDS-PAGE and identified by western blot analysis (data not shown), and proteins were quantified by Bradford protein assay. Expression of pol β was poor when the p.P261L mutation was included in the protein. Thus, p.P261L, p.P261L/T292A, p.P261L/I298T, and triple mutant were partially purified to 49, 60, 50 and 51% homogeneity, respectively. The lack of any polymerase or exonuclease activity after prolonged incubations at 37°C in the stability measurements described below (Fig. 2) indicates that *E. coli* polymerases do not likely contribute to product formation in these preparations at room temperature or 37°C and that our purification protocol removes contaminating activities from our enzyme preparations. In contrast, the WT, p.T292A, p.I298T and p.T292A/I298T variants were greater than 90% homogenous (19 and data not shown).

Following enzyme purification, we performed assays of pol β activity by using a DNA substrate that was previously used for pol β fidelity studies (25). We determined pol β catalytic efficiency based on kinetic analyses of single-nucleotide addition opposite template dG with single-nucleotide gapped DNA substrate. Since the catalytic efficiency (*k*_cat_/*K*_m,dCTP_) of the triple mutant on a gapped DNA substrate was dramatically reduced at 37°C (Fig. 2) and it was difficult to purify the triple mutant to more than 50% homogeneity, we hypothesized that this mutant may be unstable at 37°C and was degraded during bacterial expression.

To probe this hypothesis, we tested the stability of WT and all single, double and triple mutant variants of pol β by examining the sensitivity of the dCTP incorporation reaction to incubation time at 37°C. Interestingly, the triple mutant, p.I298T, p.P261L/I298T and p.T292A/I298T variants are unstable at 37°C: after 3 min of pre-incubation at 37°C, the activity of those variants was dramatically decreased (Fig. 2A–C). In contrast, the p.P261L/T292A variant and its respective single mutant variants are stable at 37°C (Fig. 2C and data not shown). These results suggest that the stability of the triple mutant at 37°C was affected specifically by the p.I298T alteration. At room temperature, the stability of the triple mutant is increased and comparable to WT (Fig. 2D). Since several of the mutant forms (e.g., p.I298T, p.P261L/I298T, p.T292A/I298T, p.P261L/T292A/I298T) do not exhibit activity at long incubation periods at 37°C, these preparations provide an opportunity to ascertain whether contaminating activities exist. The lack of any activity indicates that *E. coli* polymerases do not contribute to product formation in these preparations at room temperature and that our purification protocol removes contaminating polymerase activities from our enzyme preparations.

### Catalytic efficiencies for correct incorporation

**Figure 3.**
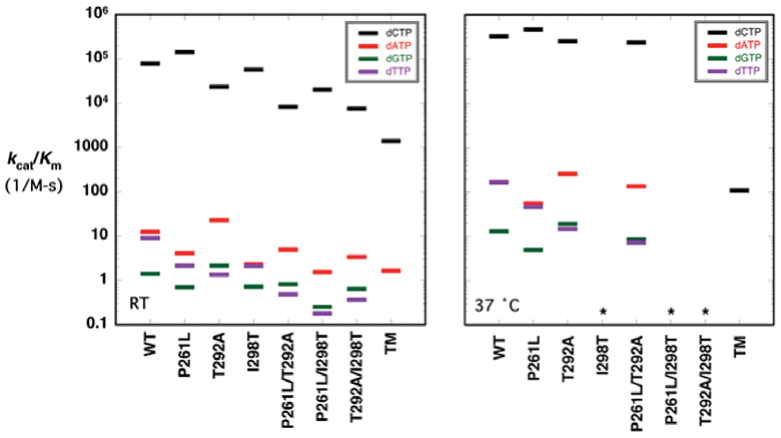
Discrimination plot for DNA polymerase β and its variants. The catalytic efficiencies for correct insertion (dCTP, black lines) and incorrect insertion (dATP, red lines; dGTP, green lines; and dTTP, purple lines) opposite a templating guanine are plotted for each enzyme. The catalytic efficiencies for room temperature (22°C, left panel) and 37°C (right panel) are taken from Tables I and II, respectively. Discrimination or fidelity is determined from the ratio of catalytic efficiencies for competing substrates (e.g., right vs. wrong nucleotide insertion). Accordingly, plots of the log of these catalytic efficiencies illustrate the difference (i.e., magnitude of discrimination/fidelity is directly proportional to the distance between the black line and the respective colored lines). The asterisks indicate that the catalytic efficiencies could not be determined due to the low stability of these variants.

Following the confirmation of decreased stability conferred by the triple mutant, we decided to test whether stability alone explains the dramatic reduction in activity at 37°C (Fig. 2), by measuring the steady-state kinetic parameters for all of the variants at room temperature (Table I; Fig. 3, left panel). As expected, the apparent *k*_cat_ values of the pol β variants that could be measured at 37°C (Table II) were lower at room temperature.

Since both the triple mutant and all I298T containing variants are unstable at 37°C, we assayed these variants at room temperature to ascertain if the modified side chains had kinetic consequences other than reducing protein stability. Variants of triple mutant, p.I298T, and p.P261L/I298T displayed a moderately reduced apparent *k*_cat_ at RT compared with WT, whereas the p.T292A/I298T variant showed similar catalytic activity to that of WT pol β at RT (Table I). Since the active fraction of enzyme is unknown in these preparations, it is difficult to come to any definitive conclusions concerning activity alone. However, the apparent *K*_m_ values are independent of active enzyme fraction and thus constitute convenient kinetic parameters to monitor altered kinetic constants (28). The apparent *K*_m,dCTP_ of the triple mutant, p.T292A/I298T, and p.P261L/I298T variants were increased at room temperature 40-, 10- and 3-fold, respectively, compared to WT (Table I). At 37°C, the apparent *K*_m,dCTP_ of the triple mutant was increased 15-fold compared to WT, similar to the change observed at RT (Table II). Likewise, the *K*_m,dCTP_ of the p.T292A and p.P261L/T292A variants were significantly increased at both RT and 37°C relative to WT (Tables I and II).

A comparison of the catalytic efficiencies or specificity constants for WT and the variants at RT indicates that the triple mutant exhibits the lowest catalytic efficiency for correct nucleotide insertion and p.P261L has similar or greater efficiency than WT (Table I; Fig. 3, left panel). The catalytic efficiencies for the other variants were intermediate between these two extremes (Table I; Fig. 3, left panel). Significantly, all p.T292A-containing variants reduced catalytic efficiency compared to WT at RT: the p.T292A variant (by 3.3-fold), the p.P261L/T292A variant (by 9.4-fold) and the p.T292A/I298T variant (by 10.4-fold) (Table I). In addition, the p.P261L/I298T variant decreased catalytic efficiency at RT by 3.9-fold (Table I). These differences are most easily seen in Fig. 3 where the catalytic efficiencies for correct insertion (dCTP, black lines) are plotted for WT and each variant (note the logarithmic ordinate scale). Therefore, the triple mutant affects catalysis independently of its effect on stability. Due to the lack of protein stability, we did not measure kinetic parameters for any of the p.I298T containing mutants at 37°C. The triple mutant displays a catalytic efficiency for correct insertion 3,000-fold lower than WT at 37°C (19; Table II; Fig. 3, right panel). The catalytic efficiency of the remaining mutants, p.P261L, p.T292A, and p.P261L/T292A was similar to that of WT at 37°C (Table II and Fig. 3, right panel).

### Fidelity of the pol β variants

**Figure 4.**
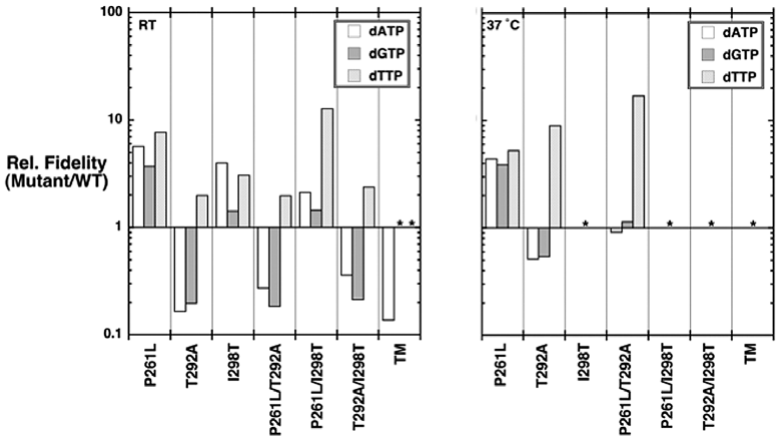
Relative fidelity of the DNA polymerase β variants. Relative fidelities (dATP, open bars; dGTP, dark grey bars; dTTP, light grey bars) opposite a templating guanine were calculated from the ratio of fidelities (variant/wild-type) determined at room temperature (22°C, left panel) and 37°C (right panel). Bars above the baseline (1) indicate an increase in fidelity and those below the baseline represent a decrease in fidelity. The asterisks indicate that the catalytic efficiencies could not be determined due to the low stability of these variants.

Misincorporation fidelity studies were performed to understand the role of the pol β variants on DNA synthesis fidelity. The apparent *k*_cat_, *K*_m,dNTP_, and catalytic efficiency (*k*_cat_/*K*_m,dNTP_) values for misincorporation at room temperature and 37°C are tabulated in Tables I and II, respectively. The instability of the p.I298T variants at 37°C precluded fidelity assays for many of the variants at the elevated temperature. The catalytic efficiencies for misinsertion are also illustrated in discrimination plots (Fig. 3) and their relative fidelities (mutant/WT) shown in Fig. 4. In discrimination plots, the distance between correct insertion (black lines) to that of a misinsertion (colored lines) is directly proportional to fidelity or misinsertion frequency (29). Accordingly, the shorter the distance between these points (correct insertion and misinsertion), the lower the fidelity. For example, focusing on the kinetic constants shown in Fig. 3 for the p.P261L mutant indicates that fidelity for all three misinsertions is greater than that for WT (e.g., efficiency for dCTP insertion is increased relative to WT and misinsertions are reduced compared to WT).

We were unable to measure the catalytic activity for dTTP and dGTP misincorporation for the triple mutant due to its very low misinsertion efficiency (even at very high enzyme concentrations). With regards to catalytic efficiency for misincorporation (*k*_cat_/*K*_m,dNTP_), the *k*_cat_/*K*_m,dNTP_ for the p.T292A variant was increased for both dATP and dGTP misincorporations and decreased for dTTP. The remaining variants showed decreased catalytic efficiencies for all misincorporations (Table I, Fig. 3). The results of misincorporation at 37°C are tabulated in Table II and illustrated in Fig. 3 (right panel). For the variants that could be examined, the results parallel those observed at room temperature (Fig. 3, left panel).

Fidelity is defined as the ratio of the sum of catalytic efficiencies of correct and incorrect nucleotide incorporation over the catalytic efficiency for misinsertion. Since the efficiency for misinsertion is much lower than that for correct insertion (Tables I and II), a simplified view is that fidelity is simply the ratio of catalytic efficiencies (correct/incorrect). As explained above, the distance between the plotted catalytic efficiencies for correct and incorrect insertions is directly proportional to fidelity (Fig. 3). The relative fidelities (mutant/WT) can easily be illustrated (Fig. 4). The triple mutant has a 7-fold lower fidelity than WT for dATP transversions at room temperature. In contrast, the p.P261L variant had a significantly higher fidelity than WT at both room temperature and 37°C. The p.I298T and p.P261L/I298T variants also exhibited a higher fidelity at room temperature for all three misinsertions that were assayed.

The p.T292A variant had decreased relative misinsertion fidelity for dATP and dGTP at room temperature and 37°C (Fig. 4), and an increased relative fidelity for dTTP at both temperatures. Thus, the p.T292A variant displays higher relative fidelity for transitions and lower relative fidelity for transversions. The p.P261L/T292A and p.T292A/I298T variants (both containing p.T292A) behave like the p.T292A variant (Fig. 4). Indeed, although the p.P261L and p.I298T variants have higher relative fidelity for dATP and dGTP misincorporation compared to the p.T292A variant, the p.T292A appears to be dominant over the other mutation in the double mutants p.P261L/T292A and p.T292A/I298T.

### DNA substrate binding

Given the dramatic effect of the triple and T292A mutations on the apparent *K*_m,dNTP_ (Tables I and II) and the structural observation that Thr292 forms a hydrogen bond with template strand (Fig. 1), we investigated its effect on the affinity for the gapped DNA substrate. A steady-state kinetic analysis indicates that DNA binding is weaker for these mutants (Table III) and that the elevated *K*_m,dNTP_ is in part due to weaker DNA binding (28). Accordingly, the poor catalytic efficiency of the triple mutant at room temperature is due to its poor DNA binding (Table III).

## Discussion

Biochemical analyses of all the single and double mutant variants that comprise the triple pol β mutant demonstrate that the loss of function shown by the triple mutant can be separated into distinct enzyme activity and stability changes. More specifically, the p.I298T pol β variant is responsible for the instability shown by the triple mutant at 37°C (Fig. 2), while the p.T292A variant is responsible for the loss of DNA synthesis fidelity (Fig. 4), and most of the reduction in catalytic efficiency seen at RT (Fig. 3, left panel). However, the triple mutant exhibits an efficiency significantly lower than that of p.T292A alone (Table I). Thus, the lower catalytic efficiency of the triple mutant represents a synergistic effect of all three mutations. The third variant, p.P261L, exhibits a modest increase in fidelity relative to WT enzyme. Interestingly, all three alterations of the triple mutant are in the same pol β subdomain (N-subdomain; Fig. 1). The N-subdomain (residues 261–335) undergoes a large conformational change upon dNTP binding to interact with the nascent base pair (2). Accordingly, alterations to this subdomain that interfere with conformational changes/adjustments or interactions with the nascent base pair would be expected to impact enzyme activity and/or fidelity.

The dramatic effect of the p.I298T pol β variant on enzyme stability observed at 37°C was surprising, but can be rationalized considering the pol β structure (Fig. 1). The hydrophobic isoleucine residue found in the wild-type enzyme would be expected to provide good packing for the interior of the N-subdomain. The threonine substitution buries a hydroxyl group that would be expected to lower the stability of this subdomain. Also noteworthy, residues 292 and 298 are adjacent to one another in anti-parallel β-strands (Fig. 1). Thus, the loss of activity at 37°C is most easily explained by the decreased stability of the N-subdomain with the p.I298T substitution. Since there is activity, albeit low, at room temperature, the N-subdomain must be at least partially folded at the lower temperature to permit interactions with the nascent base pair. Previous work had demonstrated that loss of the Arg283 interaction with the template strand by site-directed mutagenesis results in a >30,000-fold loss in catalytic efficiency (30,31). In light of such a dramatic effect, the decrease in catalytic efficiency afforded by the triple mutant is in reasonable context.

Although the p.I298T variants are unstable at 37°C, they exhibit significant activity at room temperature permitting kinetic characterization (Table I). While the activity of the pol β variants assayed at RT was similar to wild-type enzyme, catalytic efficiency was significantly decreased for the triple mutant and all p.T292A-containing variants (Table I; Fig. 3, left panel), mostly due to a significant increase in the apparent *K*_m,dNTP_ (Table I). The *K*_m,dNTP_ can increase due to an increase in the dissociation constant *K*_d,dNTP_, decreased rate of dNTP insertion, or decreased DNA binding affinity (i.e., increased dissociation rate constant, *k*_off_) (28). The p.T292A substitution is predicted to eliminate an important hydrogen bond with the DNA backbone immediately upstream of the templating (coding) nucleotide (Fig. 1). The observed increase in *K*_m,dNTP_ for the p.T292A variants may thus be partially due to decreased DNA binding affinity and is consistent with the observed increased catalytic activity of these variants (Table I) since increasing the DNA dissociation rate constant can enhance catalytic cycling (32). Consistent with this idea, the *K*_m,DNA_ for the T292A variant and triple mutant are increased relative to wild-type enzyme (Table III). However, this interpretation must be tempered since the active enzyme fraction is not known. An alternate explanation is that the loss of the hydrogen bond destabilizes the coding templating base making it more difficult for the polymerase to identify its correct base pairing partner. This could result in both an increase in the binding affinity for the incoming nucleotide and/or a decrease in the rate of insertion.

Since DNA synthesis fidelity approximates the ratio of catalytic efficiencies for right and wrong nucleotides, these efficiencies must be differentially altered for a variant polymerase to exhibit an altered fidelity. There are several examples where mutations distant from the pol β active site have impacted DNA synthesis fidelity (2). The only single nucleotide alteration of the triple mutant that directly contacts the DNA substrate, p.T292A, exhibits a significantly lower fidelity for transversions (dATP and dGTP misinsertions opposite dG; Figs. 3 and 4). Importantly, the double mutants containing p.T292A (p.P261L/T292A and p.T292A/I298T) also exhibit the transversion over transition bias. The triple mutant likewise exhibits a lower fidelity than WT for dATP misinsertion opposite G. Interestingly, the p.P261L single mutant has a significantly higher fidelity than WT at room temperature and 37°C, but the p.P261L/T292A double mutant does not. These data suggest that the p.T292A mutation is dominant compared to both p.P261L and p.I298T with regards to fidelity. Several previously characterized pol β mutants, including some found in tumors, exhibit a base substitution bias. For example, the p.D246V pol β mutant, present in the flexible loop in the catalytic C-subdomain (Fig. 1), shows a preferential misinsertion of dTTP opposite guanine relative to WT (33). This misincorporation bias makes the p.D246V enzyme a mutator mainly for C to T transitions. In contrast, all of the remaining pol β mutants we identified in prostate tumors do not exhibit this bias (19). However, fidelity is dependent on DNA sequence and dNTP pool imbalances, so a strict correlation between *in vitro* polymerase fidelity measurements with altered polymerases and their cellular impact on mutagenesis is qualitative.

Crystallographic analyses of pol β have shown that productive binding of pol β to both gapped and nicked DNA template requires a 90° bend in the DNA template (12,13). This 90° bend allows α-helix N residues of the N-subdomain of pol β to interact with the nascent base-pair in the closed conformation (Fig. 1). Various mutagenesis studies have shown that the pol β/dNTP contacts produced by this 90° bend are important for both polymerization efficiency and fidelity (10,30,32). Thus, residues that influence the equilibrium between the open and closed forms could potentially modulate enzyme activity or fidelity if they were modified. Thr292 is far from the template strand in the open binary DNA complex, but as discussed above, the hydrogen bond between Thr292 and the template strand would be expected to stabilize the closed pol β form as well as assist template base positioning. Consistent with this prediction, removal of this hydrogen bond through alanine substitution (p.T292A) results in increases of both the apparent *K*_m_ for the DNA and the incoming dNTP. Curiously, Pro261 is situated at the boundary between the C- and N-subdomains, at a position critical for the transition from open to closed form (2,13). The observation that leucine substitution of Pro261 increases fidelity of the mutant due to a decrease in catalytic efficiency for misinsertions (Fig. 3) suggests that the closed conformation is destabilized in the p.P261L mutant during incorrect nucleotide incorporation.

In summary, the *in vitro* kinetic analyses presented here demonstrate that the loss of function shown by the pol β early onset prostate tumor-associated triple mutant can be separated into distinct changes in catalytic efficiency and fidelity (primarily the p.T292A point mutation) as well as an enzyme stability defect (p.I298T mutation). The results explain the mechanistic underpinning of the dramatic loss in enzymatic activity of this tumor-associated mutant form of pol β. The fact that the wild-type allele of pol β was not found in the tumor indicates that this tumor was pol β null. This genotype would be expected to result in a base excision repair defect and thus genomic accumulation of single base lesions. Alternatively, a less faithful DNA polymerase may fulfill gap-filling DNA synthesis during repair. Previously it was shown that the loss of pol β function in mouse embryonic fibroblasts results in an increase in spontaneous and alkylation-induced mutation frequency (34). The therapeutic implications of these data are interesting to consider.

## Figures and Tables

**Table I.** Kinetic summary for single-nucleotide gap-filling opposite a templating guanine at room temperature (22°C) by wild-type pol β and variants.

Enzyme	dNTP	*k*_cat_^a^ *10^−2^ s^−1^*	*K*_m,dNTP_ *μ*M	*k*_cat_/*K*_m_ *10^−2^ μ*M*^−1^s^−1^*
WT^b^	dCTP	3.24 (0.27)	0.42 (0.04)	771 (150)
p.P261L		5.57 (0.33)	0.39 (0.04)	1,430 (80)
p.T292A		4.98 (0.13)	2.16 (0.16)	231 (11)
p.I298T		1.35 (0.30)	0.24 (0.03)	563 (51)
p.P261L/T292A		4.98 (0.52)	6.06 (0.20)	82 (8)
p.P261L/I298T		2.50 (0.27)	1.25 (0.10)	200 (27)
p.T292A/I298T		3.25 (0.08)	4.38 (0.35)	74 (9)
TM^c^		2.36 (0.34)	17.08 (4.48)	14 (1)
WT	dATP	0.164 (0.012)	131 (22)	0.125 (0.029)
p.P261L		0.135 (0.005)	331 (14)	0.041 (0.001)
p.T292A		0.223 (0.010)	98 (11)	0.227 (0.015)
p.I298T		0.091 (0.006)	397 (64)	0.023 (0.003)
p.P261L/T292A		0.095 (0.001)	195 (21)	0.049 (0.008)
p.P261L/I298T		0.037 (0.005)	244 (44)	0.015 (<0.001)
p.T292A/I298T		0.125 (0.018)	373 (60)	0.035 (0.002)
p.TM		0.046 (0.002)	282 (27)	0.016 (0.002)
WT	dGTP	0.110 (0.007)	787 (55)	0.014 (0.001)
p.P261L		0.211 (0.069)	3,036 (1,397)	0.007 (0.001)
p.T292A		0.113 (0.009)	531 (44)	0.021 (<0.001)
p.I298T		0.064 (0.006)	880 (262)	0.007 (0.001)
p.P261L/T292A		0.024 (0.003)	293 (66)	0.008 (0.001)
p.P261L/I298T		0.055 (0.010)	2,205 (589)	0.003 (<0.001)
p.T292A/I298T		0.019 (0.002)	293 (80)	0.006 (0.002)
TM		ND^d^	ND	ND
WT	dTTP	0.244 (0.008)	275 (52)	0.089 (0.013)
p.P261L		0.143 (0.017)	665 (65)	0.021 (0.001)
p.T292A		0.110 (0.021)	822 (164)	0.013 (0.001)
p.I298T		0.089 (0.026)	424 (188)	0.021 (0.003)
p.P261L/T292A		0.017 (0.001)	367 (75)	0.005 (0.001)
p.P261L/I298T		0.019 (0.002)	1,068 (77)	0.002 (<0.001)
p.T292A/I298T		0.017 (0.003)	483 (102)	0.004 (0.001)
TM		ND	ND	ND

The results represent the mean (SD) of at least three independent determinations.

a Apparent *k*_cat_ was calculated from *V*_max_, where *k*_cat_ = *V*_max_/(apparent enzyme). The apparent enzyme concentration was estimated from total protein.

b Wild-type human DNA polymerase β.

c Triple mutant (p.P261L/T292A/I298T) of human DNA polymerase β.

d Not determined.

**Table II.** Kinetic summary for single-nucleotide gap-filling opposite a templating guanine at 37°C by wild-type pol β and variants.

Enzyme	dNTP	*k*_cat_^a^ *10^−2^ s^−1^*	*K*_m,dNTP_ *μ*M	*k*_cat_/*K*_m_ *10^−2^ μ*M*^−1^s^−1^*
WT^b^	dCTP	8.09 (2.09)	0.25 (0.10)	3,240 (920)
p.P261L		13.6 (0.9)	0.29 (0.07)	4,690 (1,450)
p.T292A		17.3 (1.2)	0.68 (0.28)	2,540 (730)
p.I298T		ND^c^	ND	ND
p.P261L/T292A		25.6 (1.2)	1.08 (0.15)	2,370 (300)
p.P261L/I298T		ND	ND	ND
p.T292A/I298T		ND	ND	ND
TM^d^		0.040 (0.003)	3.72 (0.17)	1.08 (0.08)
WT	dATP	0.786 (0.012)	47 (2)	1.680 (0.093)
p.P261L		0.630 (0.024)	114 (5)	0.553 (0.026)
p.T292A		0.846 (0.048)	33 (2)	2.557 (0.032)
p.I298T		ND	ND	ND
p.P261L/T292A		0.792 (0.037)	59 (4)	1.348 (0.019)
p.P261L/I298T		ND	ND	ND
p.T292A/I298T		ND	ND	ND
TM		ND	ND	ND
WT	dGTP	0.587 (0.001)	446 (16)	0.131 (0.005)
p.P261L		0.527 (0.062)	1,069 (223)	0.049 (0.005)
p.T292A		0.709 (0.032)	376 (42)	0.189 (0.015)
p.I298T		ND	ND	ND
p.P261L/T292A		0.490 (0.017)	586 (25)	0.084 (0.001)
p.P261L/I298T		ND	ND	ND
p.T292A/I298T		ND	ND	ND
TM		ND	ND	ND
WT	dTTP	0.870 (0.012)	52 (2)	1.667 (0.030)
p.P261L		0.702 (0.028)	152 (15)	0.460 (0.030)
p.T292A		0.715 (0.048)	491 (43)	0.146 (0.005)
p.I298T		ND	ND	ND
p.P261L/T292A		0.410 (0.011)	569 (34)	0.072 (0.003)
p.P261L/I298T		ND	ND	ND
p.T292A/I298T		ND	ND	ND
TM		ND	ND	ND

The results represent the mean (SD) of at least three independent determinations.

a Apparent *k*_cat_ was calculated from *V*_max_, where *k*_cat_ = *V*_max_/(apparent enzyme). The apparent enzyme concentration was estimated from total protein.

b Wild-type human DNA polymerase β.

c Not determined due to poor stability of the protein.

d Triple mutant (p.P261L/T292A/I298T) of human DNA polymerase β.

**Table III.** Dependence of DNA synthesis on single-nucleotide gapped DNA concentration at room temperature and 37°C by wild-type pol β and variants.

Enzyme	Temperature	*k*_cat_^a^ *10^−2^ s^−1^*	*K*_m,DNA_ *μ*M	*k*_cat_/*K*_m_ 10^−2^ *μ*M*^−1^s^−1^*
WT^b^	RT	7.8 (0.2)	0.16 (0.01)	4,875 (75)
	37°C	9.8 (0.5)	0.07 (<0.01)	14,000 (240)
TM^c^	RT	40.4 (2.0)	1.5 (0.1)	2,700 (20)
	37°C	ND^d^	ND	ND
T292A	RT	3.6 (0.1)	0.25 (0.02)	1,440 (40)
	37°C	38.6 (7.3)	1.48 (0.32)	2,610 (60)

The results represent the mean (SD) of at least three independent determinations. The templating base in the gap was guanine and the dCTP concentration was 100 *μ*M.

a Calculated from total protein concentration.

b Wild-type human DNA polymerase β.

c Triple mutant (P261L/T292A/I298T) of human DNA polymerase β.

d Not determined.
